# A universal DNA microarray for rapid fish species authentication

**DOI:** 10.1016/j.fochms.2025.100241

**Published:** 2025-01-19

**Authors:** Patrizia Bade, Sebastian Stix, Kristina Kappel, Jan Fritsche, Ilka Haase, Andrew Torda, Nils Wax, Markus Fischer, Dirk Brandis, Ute Schröder

**Affiliations:** a National Reference Centre for Authentic Food, Max Rubner-Institut (MRI), Hermann-Weigmann-Str. 1, 24103 Kiel, Germany; b Department of Safety and Quality of Milk and Fish Products, Max Rubner-Institut (MRI), Hermann-Weigmann-Str. 1, 24103 Kiel, Germany; c National Reference Centre for Authentic Food, Max Rubner-Institut (MRI), E.-C.-Baumann-Str. 20, 95326 Kulmbach, Germany; d Centre for Bioinformatics, University of Hamburg, Albert-Einstein-Ring 8, 22761 Hamburg, Germany; e Hamburg School of Food Science, Institute of Food Chemistry, University of Hamburg, Grindelallee 117, 20146 Hamburg, Germany; f Zoological Museum of the University of Kiel, Hegewischstraße 3, 24105 Kiel, Germany

**Keywords:** DNA chip, Food fraud, Universal DNA probes, DNA hybridization, Screening method, *In silico*

## Abstract

DNA microarrays are now used in fields such as gene expression analysis, pathogen/virus detection and identification of biomarkers. Although they have been used in the food sector for species identification, they detect a limited number of species and are thus less suited for fishery products due to the large variety of traded species. Here, the aim of this proof-of-principle study was to design a universal DNA microarray that should be able to distinguish all fish species by comparing hybridization signal patterns from samples with patterns obtained from reference specimens. A universal set of 100 DNA probes (based on the genetic marker genes 16S ribosomal RNA and cytochrome *b*) generated species-specific DNA probe patterns for all 86 analyzed fish specimens. This new screening method shows potential to authenticate specimens from all fish species and by this could play an important role in fighting fraudulent practices and adulteration in the seafood sector.

## Highlights

• A universal DNA microarray for rapid fish species identification was designed.
• All DNA probes generated species-specific signal patterns
• 86 specimens from 35 fish species in 14 families were distinguished.
• The DNA chip identified fillet market samples at species level.

## Graphical abstract

**Figure:**
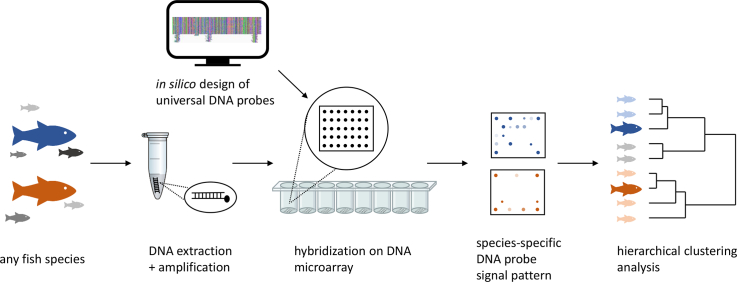

## 1 Introduction

The global fish trade increases steadily each year. Aquaculture production as well as the number of fisheries has expanded greatly in the last decades, reaching 178 million tons in 2020 (FAO, 2022a). In parallel, global aquatic food consumption increased at an average annual rate of 3 % (FAO, 2022b). Long, branched supply chains make traceability of these goods challenging. Moreover, morphological species identification of processed products (*e.g.* fish fillets) is often very difficult. In order to ensure better monitoring of production chains and detect food fraud and adulteration in fish, European legislation introduced detailed requirements for the labelling of fisheries and aquaculture products (Regulation (EU) No. 1379/2013). Still, fish species substitutions happen quite often, as seafood is one of the top 10 most adulterated foods (ACN, 2023). In order to stop incorrectly labelled fish products reaching the market, trading companies need rapid tests to authenticate fishery goods as part of quality management programs.

Much attention has focused on the development of molecular techniques to determine the identity and authenticity of fish products. DNA barcoding has been suggested as the gold standard in species verification (Dawan & Ahn, 2022; Filonzi et al., 2023). Both nuclear and mitochondrial gene markers are used in diverse approaches (Elyasigorji, Izadpanah, Hadi, & Zare, 2022). However, a relatively high mutation rate and DNA copy number as well as a historical basis make mitochondrial gene markers effective for species identification. 16S ribosomal rRNA gene (16S rDNA) (Horreo, Fitze, Jiménez-Valverde, Ari Noriega, & Pelaez, 2019), cytochrome *b* gene (*cytb*) (Barbosa, Sampaio, da Silva, Leite Alcântara, & Santos, 2020) and cytochrome *c* oxidase subunit I gene (COI) (Sultana, Ali, Hossain, Asing Naquiah, & Zaidul, 2018) are widely used. Because the analysis of DNA sequence data requires a lot of expertise, multiple service laboratories provide DNA barcoding. Moreover, this analysis is comparatively expensive and it usually takes a few days for companies to receive the results of the species verification, which is unacceptable, especially for fresh products. Affordable, rapid and user-friendly fish species tests are urgently needed.

Several rapid and cost-effective DNA-based methods for fish species identification have been developed. Loop-mediated isothermal amplification (But, Wu, Shao, & Shaw, 2020; Li, Kong, Xie, Yu, & Chen, 2022; Spielmann et al., 2019; Wax et al., 2023), real-time PCR (Bojolly et al., 2017), paper-based lateral flow assay (Jauset-Rubio et al., 2016) and multiplex PCR assays (Lee, Suh, Lee, & Kim, 2022; Trotta et al., 2005) are available for individual fish species. DNA microarrays may offer an effective alternative. They are used in diverse fields: detection and differentiation of pathogens and viruses (Jin, Li, & Xu, 2019; Xiang et al., 2022), identification of biomarkers and genes (Xiao, Wu, Dang, & Ju, 2018), food allergens (Tortajada-Genaro et al., 2011) and, of course, gene expression (Furihata & Suzuki, 2023; Shirai, Shimoda, Takahashi, Takayama, & Kikuchi, 2023). Commercially available DNA chips for the detection of animal specimens (mammals and birds) are used in official food surveillance laboratories (Verrez-Bagnis et al., 2018) and even a DNA microarray was developed to identify five marine mammal species in feed and food (Huan, Zhang, He, & Zhang, 2021).

In a previous study, a user-friendly and rapid colorimetric DNA chip was developed for the authentication of ten important food fish species (Kappel, Eschbach, Fischer, & Fritsche, 2020) and two crustacean species (Kappel, Fafińska, Fischer, & Fritsche, 2021). The analysis required four to five hours of work. 67 different fish species and 47 crustacean species were tested and grouped in species-specific clusters by comparing the DNA probe signal patterns of all species. Unfortunately, the method was only designed for 10 or 12 target species.

The aim of this proof-of-principle study was to test whether a universal DNA microarray could be designed that is able to distinguish all food-relevant fish species. Therefore, a set of 100 oligonucleotide probes was designed that could allow the species identification of unknown fish samples based on comparison of the generated probe signal patterns with those of reference specimens. This should provide a rapid helpful molecular tool for service laboratories and official food control authorities.

## 2 Materials and methods

### 2.1 DNA microarray design

The universal DNA microarray consists of a set of 100 DNA probes. 96 universal DNA probes were designed in an automated approach to bind to regions on 16S rDNA and *cytb* (see 2.2 and 2.3). Four additional probes from a previous study were chosen as control probes (Kappel, 2009; Kappel et al., 2020): DNA probe uni_16S_05 from the conserved region of the 16S rDNA target segment was designated as a positive control and NK02 as negative control probe (no sequence match for any fish species *via* BLAST in GenBank (Basic Local Alignment Search Tool)). The probe PK01 was selected as hybridization control to target a synthetic oligonucleotide and the probe PK02 was designated as PCR amplification control of pUC57 vector DNA (ThermoFisher, Waltham, USA) (Kappel, 2009).

### 2.2 DNA probe design

Several programs and settings were used for DNA probe design: MAFFT 7.505 was used for all sequence alignments (Katoh & Standley, 2013). BLAST was used for sequence database searches against GenBank (Sayers et al., 2022), which was also used as the source for mitochondrial genomes (Benson, Karsch-Mizrachi, Lipman, Ostell, & Sayers, 2010). Given a set of aligned sequences and a similarity matrix from MAFFT, “reduce” was used to extract representative sequences which span the sequence space of the aligned set as evenly as possible (Torda, 2020). Sequence entropy (variability) was calculated from $S=\Sigma_{a=1}^{4}\;p_{a}\log_{4}\left(p_{a}\right)$ for each column in an alignment (Sander & Schneider, 1991). $p_{a}$ is the probability/frequency of a base type a in the column and the summation runs over the four types of bases. Gaps were treated as missing data. The Vienna Suite v2.6.0 was used for all calculations of free energy of hybridization and melting temperature (Cock et al., 2009; Lorenz et al., 2011; SantaLucia, 1998). The find peaks function in the signal processing module of sci-py was used with default values to locate regions of maximum and minimum sequence variation (Virtanen et al., 2020).

**Fig. 1:**
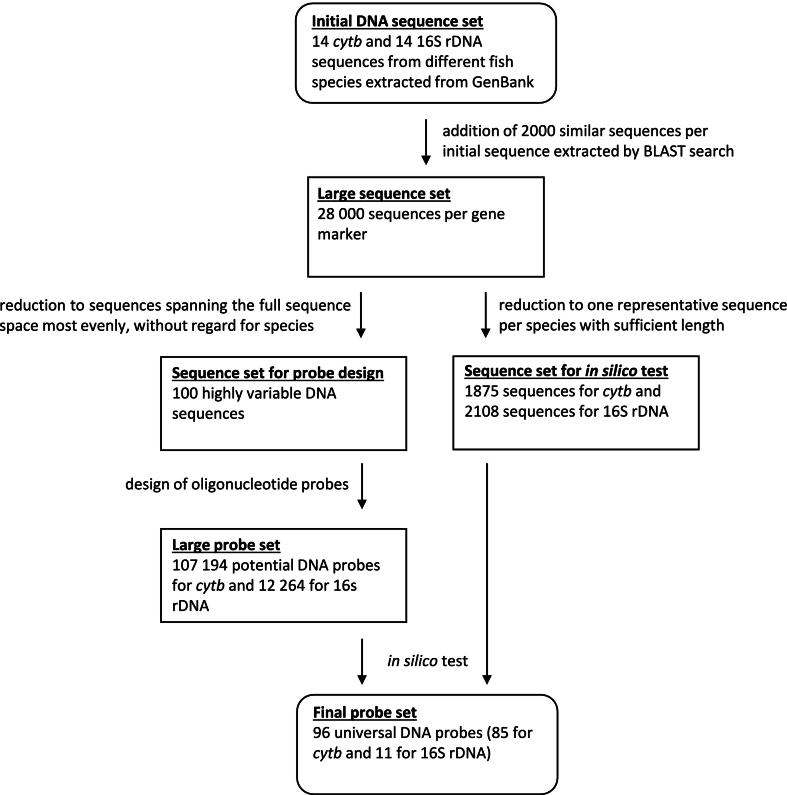
Overview of the procedure of designing 96 universal DNA probes (for details see 2.2 and 2.3)

A universal probe set for the discrimination of fish species was designed according to a novel multistep procedure (compare with Fig. 1). First, complete mitochondrial DNA sequences for 14 fish species were retrieved from GenBank (Benson et al., 2010), which showed the most abundant haplotype/DNA sequence, in particular: *Solea senegalensis* (Senegalese sole, NC_008327.1), *Gadus chalcogrammus* (Alaska pollock, AM489719.1), *Gadus morhua* (Atlantic cod, NC_002081.1), *Pangasianodon hypophthalmus* (striped catfish, MZ286355.1), *Katsuwonus pelamis* (skipjack tuna, JN086155.1), *Clupea harengus* (Atlantic herring, KC193692.1), *Oncorhynchus mykiss* (rainbow trout, NC_001717.1), *Scomber colias* (Atlantic chub mackerel, NC_013724.1), *Diplodus sargus* (white seabream, NC_057561.1), *Lutjanus vitta* (brownstripe red snapper, NC_042930.1), *Dicentrarchus labrax* (European seabass, NC_026074.1), *Salmo salar* (Atlantic salmon, NC_001960.1), *Hemigymnus melapterus* (blackeye thicklip, NC_057241.1) and *Aesopia cornuta* (unicorn sole, NC_021969.1). Sequences were truncated according to the target sequence regions of interest of *cytb* (approx. 464 bp) and 16S rDNA (approx. 600 bp) defined by the primer sequences (see 2.6) (“Initial DNA sequences set”). A database search was run with each of the 28 sequences (1 per gene marker and species) and the first 2000 similar sequence segments (limited to Actinopterygii and Chondrichthyes) were retrieved (“Large sequence set”). Each set of 28,000 sequences per gene marker were aligned and the set of 100 sequences were extracted which most evenly spanned the full sequence space, without regard for species (“Sequence set for probe design”). This set of 100 sequences was then used to search for conserved or variable regions to design a pool of candidate DNA probes.

Sequence entropy was calculated from the alignment and the four maxima (*cytb*) and three minima (16S rDNA) were located and stored. The set of 100 sequences was then used to construct two large pools of candidate probes per gene marker. Starting from the 5′ end, all unique segments m were collected from each of the 100 sequences. m was taken as 15, 16, 17, 18, 19 and 20 nucleotides, giving sets of 107,194 fragments for *cytb* and 12,264 for 16 s rDNA (“Large probe set”). This massive candidate pool was then filtered using *ad hoc* rules based on literature experience (Li, He, & Zhou, 2005). The fragment must (a) include one of the ten minima/maxima sequence entropy locations, (b) not have more than three G or three C in the 5′ last five bases, (c) have GC content between 35 and 65 %, (d) not have more than four adjacent repeated bases, (e) have a predicted melting temperature between 56.5 and 63.5 °C and (f) bind to a member of a test sequence set (for details see 2.3) with a predicted free energy of hybridization stronger than −11.5 kcal/mol, at least 9 matching bases and 85 % complementary sequence identity. The criteria were applied successively, so the number of energy calculations was large, but manageable. Finally, the candidates were sorted by the number of hybridizations and the candidate with the highest number of hybridizations for each species were picked. If all species from the test set potentially hybridize to at least one candidate, candidates with highest number of hybridizations for underrepresented species were chosen. The best 85 (*cytb*) and 11 (16S rDNA) DNA probes were kept (“Final probe set”) (Table 1 and Table B.1).

**Table 1:** Sequences and characteristics of all 100 universal DNA probes (96 newly designed DNA probes and four control probes (Kappel, 2009)).

DNA probe	Target	Sequence (5′-3′)	Length (nt)	GC content (%)	T_m_ (°C)
16s_01	16S rDNA	AGGGTCTTCTCGTCTT	16	50	58.1
16s_02	16S rDNA	CCAACATCGAGGTCGTAAA	19	47	63.0
16s_03	16S rDNA	ACATCGAGGTCGTAAGC	17	53	61.3
16s_04	16S rDNA	AACATCGAGGTCGTAAACT	19	42	62.0
16s_05	16S rDNA	GAACATCGAGGTCGTAAA	18	44	59.6
16s_06	16S rDNA	AGGGTCTTCTCGTCTTG	17	53	60.4
16s_07	16S rDNA	ATACCGCGGCCGTTT	15	60	63.5
16s_08	16S rDNA	AAGGGTCTTCTCGTCTT	17	47	59.5
16s_09	16S rDNA	AATACCGCGGCCGTT	15	60	63.5
16s_10	16S rDNA	TCCAACATCGAGGTCGTA	18	50	63.0
16s_11	16S rDNA	AGGGTCTTCTCGTCTA	16	50	57.0
cytb_01	cytb	ACGGATGAGAATGCTGTG	18	50	62.7
cytb_02	cytb	ACGGATGAGAATGCTGT	17	47	60.8
cytb_03	cytb	TACGGATGAGAATGCTGT	18	44	61.0
cytb_04	cytb	ACGGATGAGAATGCTG	16	50	58.1
cytb_05	cytb	GGATGAGAAGGCGGTA	16	56	59.6
cytb_06	cytb	GGGGAGGTCAACTAGT	16	56	58.6
cytb_07	cytb	AGGTCGACTAGTGCA	15	53	57.5
cytb_08	cytb	GGAGGTCGACTAGTGCA	17	59	63.0
cytb_09	cytb	TGATGAGAAGGCTGTT	16	44	56.5
cytb_10	cytb	ACGAAGGCTGTTATTATGA	19	37	59.4
cytb_11	cytb	TACGAAGGCTGTTATTATGA	20	35	59.6
cytb_12	cytb	CTACGAAGGCTGTTATTATG	20	40	59.2
cytb_13	cytb	ACGGATGAGAAGGCTA	16	50	58.3
cytb_14	cytb	GGAGGAGAAGGCTGTT	16	56	59.8
cytb_15	cytb	TCAACTAGTGCGTCGTTT	18	44	62.1
cytb_16	cytb	GAGGAGAAGGCTGTG	15	60	56.9
cytb_17	cytb	ACTAGTGCGTCGTTG	15	53	57.3
cytb_18	cytb	GGGAGGTCTACTAATGC	17	53	58.0
cytb_19	cytb	TGGGAGGTCTACTAATGC	18	50	60.7
cytb_20	cytb	CGAAAGCTGTTATTATTACG	20	35	58.0
cytb_21	cytb	AGGTCTACTAGTGCGTCA	18	50	62.3
cytb_22	cytb	TGTGGCGATGTCGGAT	16	56	63.1
cytb_23	cytb	GCAGTTATTATGACAAGGAG	20	40	59.4
cytb_24	cytb	ACGGAGGAGAAAGCG	15	60	59.7
cytb_25	cytb	CCTGTGAGGATTTGGA	16	50	57.0
cytb_26	cytb	GGTCAATTAATGCGTTGTTT	20	35	61.1
cytb_27	cytb	GAGGTCAATTAATGCGTTGT	20	40	62.4
cytb_28	cytb	GGAGGTCAATTAATGCGTTG	20	45	63.1
cytb_29	cytb	GGGAGGTCAATTAATGCG	18	50	61.1
cytb_30	cytb	GGGAGGTCAATTAATGC	17	47	57.3
cytb_31	cytb	GGGAGATCGACTAGTG	16	56	56.6
cytb_32	cytb	AACGAAGGCTGTTATTATGA	20	35	60.5
cytb_33	cytb	CCGGTGAGGATTTGGA	16	56	60.3
cytb_34	cytb	ACGAAGGCAGTTATTATTGT	20	35	60.8
cytb_35	cytb	CACGAAGGCAGTTATTATTG	20	40	60.3
cytb_36	cytb	CCTGTTAGGATTTGAGCAA	19	42	60.7
cytb_37	cytb	GAGAAGGCTATGGAAGTGTC	20	50	63.4
cytb_38	cytb	ACGAAGGCAGTTATTATAAC	20	35	58.7
cytb_39	cytb	CGAAGGCTGTTATTATTACC	20	40	59.5
cytb_40	cytb	GAGAAGGCTGTTTCAATGTC	20	45	62.7
cytb_41	cytb	CGGATGAGAAGGCTGTT	17	53	61.6
cytb_42	cytb	TACGAAGGCAGTCATTATTG	20	40	61.2
cytb_43	cytb	GAAGGCTGTTATTATGACAA	20	35	58.9
cytb_44	cytb	GAAGGCTGTTATTATGACA	19	37	57.8
cytb_45	cytb	CGAAGGCTGTTATTATGACA	20	40	61.2
cytb_46	cytb	GGATGAGAAAGCTGTCTGAA	20	45	63.3
cytb_47	cytb	AACGAAGGCTGTTATTATCA	20	35	60.5
cytb_48	cytb	ACGAAGGCTGTTATTATC	18	39	56.9
cytb_49	cytb	AACGAAGGCTGTTATTATC	19	37	58.2
cytb_50	cytb	CAACGAAGGCTGTTATTATC	20	40	60.0
cytb_51	cytb	GGGGAGGTCAATTAGG	16	56	57.2
cytb_52	cytb	GCAGTTATTATTACAAGGAG	20	35	56.9
cytb_53	cytb	GGAGGTCGATGAGTG	15	60	56.6
cytb_54	cytb	GGGAGGTCGATGAGT	15	60	58.1
cytb_55	cytb	TGATGAGAAGGCGGTT	16	50	59.8
cytb_56	cytb	GTCAATTAGGGCGTCGTT	18	50	63.0
cytb_57	cytb	GCGGTTGAGATGTCTGAT	18	50	62.4
cytb_58	cytb	GGATGAGAAGGCGGTT	16	56	60.7
cytb_59	cytb	ACGGATGAGAAGGCA	15	53	58.4
cytb_60	cytb	GGTCAATTAGTGCGTTGTTT	20	40	62.9
cytb_61	cytb	GAGGTCAATTAGTGCGTT	18	44	60.2
cytb_62	cytb	GGAGGTCAATTAGTGCGTT	19	47	63.3
cytb_63	cytb	GTGACGGATGAAAAGGCA	18	50	63.3
cytb_64	cytb	ACTAGTGCGTCGTTC	15	53	56.9
cytb_65	cytb	GACTAGTGCGTCGTTC	16	56	58.9
cytb_66	cytb	GAAGGCTGTAGCGATGTC	18	56	63.1
cytb_67	cytb	GAGAAGGCTGTAGCGATG	18	56	62.8
cytb_68	cytb	TGAGAAGGCTGTAGCGAT	18	50	63.4
cytb_69	cytb	TGAGAAGGCTGTAGCGA	17	53	62.6
cytb_70	cytb	CTACAAAGGCAGTTATTATG	20	35	57.2
cytb_71	cytb	CGAAGGCGGTTATTATC	17	47	57.2
cytb_72	cytb	CGGTTATTATTACTAGGAGA	20	35	56.6
cytb_73	cytb	GCGGTTATTATTACTAGGAG	20	40	58.3
cytb_74	cytb	GAAGGCGGTCATTATTACAA	20	40	61.5
cytb_75	cytb	CCTGTTAGGATTTGGGTGA	19	47	62.3
cytb_76	cytb	AACGAAGGCAGTTATTATAG	20	35	58.4
cytb_77	cytb	CGTTGCGATTTGGGTAA	17	47	60.4
cytb_78	cytb	GAAGGCTGTTGAGATGTCA	19	47	63.0
cytb_79	cytb	GGTAGGTCAACTAGTGCA	18	50	61.2
cytb_80	cytb	GGAGGTCAATTAGTGCAC	18	50	60.6
cytb_81	cytb	GGGAGGTCAATTAGTGCA	18	50	62.0
cytb_82	cytb	TGGGAGGTCTACGAG	15	60	57.2
cytb_83	cytb	AAGGCGGTTATTATCACGAG	20	45	63.5
cytb_84	cytb	GACAAAGGCGGTTATTATC	19	42	59.2
cytb_85	cytb	CGACAAAGGCGGTTATTATC	20	45	62.5
NK02		ATATTCTGCCCGCAGTTA	18	44	61.4
PK01	PK-Oligo	CGGATAACAATTTCACACAGT	21	38	62.7
PK02	pUC57–542-mer	AGTTGGCCGCAGTGTTA	17	53	64.3
uni_16s_05	16S rDNA	TTACGACCTCGATGTTGG	18	50	62.0

### 2.3 Sequence set for *in silico* test

Potentially the most time-consuming criterion in the list above (f) required the calculation of hybridization free energy using a set of sequences to model a real selection of sequences that might occur in practice. For this purpose, a fish DNA sequence set was established based on the previous 28,000 sequences (see 2.2). Only sequences of sufficient length (460 nucleotides for *cytb* and 615 nucleotides for 16S rDNA) were kept and clustered by species. A consensus sequence for each species cluster was determined, and the most similar sequence was selected as the representative sequence for the sequence set. This left 1875 representative sequences for *cytb* and 2108 sequences for 16S rDNA in the test set (“Sequence set for *in silico* test”).

### 2.4 Collection of fish specimens

Samples from deceased fish specimens (obtained as fish fillets or fish products produced for the food supply chain) (Table 2) were mainly sourced from previous projects or provided by German seafood traders and processors, the German food control authority, the James Cook University Australia or were purchased at local fishmongers and supermarkets. The species of all samples was confirmed by conventional PCR and Sanger sequencing of *cytb* and/or COI (DIN, 2019). If it was not possible to clearly identify the species, all listed names of the genus are given.

**Table 2:** Information on fish sample materials.

Usage	Sample #	Family	Genus	Species	Original sample name
Reference samples	1	Gadidae	*Gadus*	*Gadus macrocephalus*	Gmac01a
	2	Gadidae	*Gadus*	*Gadus macrocephalus*	Gmac01b
	3	Gadidae	*Gadus*	*Gadus macrocephalus*	Gmac01c
	4	Gadidae	*Gadus*	*Gadus morhua*	Gmor01a
	5	Gadidae	*Gadus*	*Gadus morhua*	Gmor02a
	6	Gadidae	*Gadus*	*Gadus morhua*	Gmor02b
	7	Gadidae	*Gadus*	*Gadus chalcogrammus*	Gchal01a
	8	Gadidae	*Gadus*	*Gadus chalcogrammus*	Gchal02a
	9	Gadidae	*Gadus*	*Gadus chalcogrammus*	Gchal02b
	10	Clupeidae	*Clupea*	*Clupea harengus*	Char04a
	11	Clupeidae	*Clupea*	*Clupea harengus*	Char05
	12	Clupeidae	*Clupea*	*Clupea pallasii*	Cpallas01a
	13	Clupeidae	*Thunnus*	*Clupea pallasii*	Cpallas01b
	14	Clupeidae	*Sprattus*	*Sprattus sprattus*	Sspratt01a
	15	Clupeidae	*Sprattus*	*Sprattus sprattus*	Sspratt01b
	16	Soleidae	*Solea*	*Solea solea*	Ssol02a
	17	Soleidae	*Solea*	*Solea solea*	Ssol02b
	18	Soleidae	*Solea*	*Solea solea*	Ssol01c
	19	Soleidae	*Solea*	*Solea senegalensis*	Ssen01a
	20	Soleidae	*Solea*	*Solea senegalensis*	Ssen01b
	21	Soleidae	*Solea*	*Solea aegyptiaca*	Saegypt02
	22	Soleidae	*Solea*	*Solea aegyptiaca*	A078
	23	Scombridae	*Thunnus*	*Thunnus albacares*	Talb01
	24	Scombridae	*Thunnus*	*Thunnus albacares*	Talb02
	25	Scombridae	*Thunnus*	*Thunnus albacares*	NRZ-Fisch-2023-001a
	26	Scombridae	*Thunnus*	*Thunnus obesus*	Tobes01a
	27	Scombridae	*Thunnus*	*Thunnus obesus*	Tobes01b
	28	Scombridae	*Thunnus*	*Thunnus obesus*	LF 7b
	29	Scombridae	*Thunnus*	*Thunnus alalunga*	Talal01a
	30	Scombridae	*Thunnus*	*Thunnus alalunga*	Talal01b
	31	Scombridae	*Thunnus*	*Thunnus alalunga*	Talal01c
	32	Scombridae	*Katsuwonus*	*Katsuwonus pelamis*	Kpel01a
	33	Scombridae	*Katsuwonus*	*Katsuwonus pelamis*	Kpel01b
	34	Scombridae	*Katsuwonus*	*Katsuwonus pelamis*	Kpel01c
	35	Lutjanidae	*Lutjanus*	*Lutjanus malabaricus*	Lmalab03
	36	Lutjanidae	*Lutjanus*	*Lutjanus malabaricus*	Lmalab04
	37	Lutjanidae	*Pinjalo*	*Pinjalo pinjalo*	Ppinj01a
	38	Lutjanidae	*Pinjalo*	*Pinjalo pinjalo*	Ppinj01b
	39	Lutjanidae	*Lutjanus*	*Lutjanus bohar*	Lboh01a
	40	Lutjanidae	*Lutjanus*	*Lutjanus bohar*	Lboh01b
	41	Lutjanidae	*Lutjanus*	*Lutjanus argentimaculatus*	Larg01a
	42	Lutjanidae	*Lutjanus*	*Lutjanus argentimaculatus*	Larg01b
	43	Salmonidae	*Salmo*	*Salmo salar*	A002
	44	Salmonidae	*Salmo*	*Salmo salar*	Ssal02a
	45	Salmonidae	*Salmo*	*Salmo salar*	Ssal01a
	46	Salmonidae	*Salmo*	*Salmo trutta*	tube 226
	47	Salmonidae	*Salmo*	*Salmo trutta*	Strutt01
	48	Salmonidae	*Salmo*	*Salmo trutta*	Strutt02
	49	Salmonidae	*Oncorhynchus*	*Onchorhynchus mykiss*	Omyk01a
	50	Salmonidae	*Oncorhynchus*	*Onchorhynchus mykiss*	Omyk03a
	51	Salmonidae	*Oncorhynchus*	*Onchorhynchus keta*	Oket02b
	52	Salmonidae	*Oncorhynchus*	*Onchorhynchus keta*	Oket02a
	53	Salmonidae	*Oncorhynchus*	*Onchorhynchus keta*	Oket01
	54	Merlucciidae	*Merluccius*	*Merluccius capensis*	A021
	55	Merlucciidae	*Merluccius*	*Merluccius capensis*	tube 166
	56	Merlucciidae	*Merluccius*	*Merluccius bilinearis*	IIM_MBIL2
	57	Merlucciidae	*Merluccius*	*Merluccius bilinearis*	IIM_MBIL3
	58	Merlucciidae	*Merluccius*	*Merluccius merluccius*	Merluc01a
	59	Merlucciidae	*Merluccius*	*Merluccius merluccius*	Merluc01b
	60	Pleuronectidae	*Pleuronectes*	*Pleuronectes platessa*	A282
	61	Pleuronectidae	*Pleuronectes*	*Pleuronectes platessa*	A283
	62	Pleuronectidae	*Pleuronectes*	*Pleuronectes platessa*	Pplat01a
	63	Pleuronectidae	*Platichthys*	*Platichthys flesus*	A294
	64	Pleuronectidae	*Platichthys*	*Platichthys flesus*	Pfles01a
	65	Pleuronectidae	*Platichthys*	*Platichthys flesus*	Pfles01b
	66	Pleuronectidae	*Pleuronectes*	*Pleuronectes quadrituberculatus*	Pqua01a
	67	Pleuronectidae	*Pleuronectes*	*Pleuronectes quadrituberculatus*	Pqua01b
	68	Pleuronectidae	*Pleuronectes*	*Pleuronectes quadrituberculatus*	Pqua01c
	69	Pangasiidae	*Pangasianodon*	*Pangasianodon hypophthalmus*	Phyp01a
	70	Pangasiidae	*Pangasianodon*	*Pangasianodon hypophthalmus*	Phyp01b
	71	Clariidae	*Clarias*	*Clarias gariepinus*	Cgar01a
	72	Clariidae	*Clarias*	*Clarias gariepinus*	Cgar01b
	73	Anguillidae	*Anguilla*	*Anguilla japonica*	tube 111
	74	Anguillidae	*Anguilla*	*Anguilla japonica*	A230
	75	Anguillidae	*Anguilla*	*Anguilla anguilla*	A231
	76	Anguillidae	*Anguilla*	*Anguilla anguilla*	tube 124
	77	Dasyatidae	*Neotrygon*	*Neotrygon orientalis*	NRZ-Fisch-2023-004
	78	Dasyatidae	*Neotrygon*	*Neotrygon orientalis*	NRZ-Fisch-2023-005
	79	Dasyatidae	*Maculabatis*	*Maculabatis macrura/gerrardi*	NRZ-Fisch-2023-006
	80	Dasyatidae	*Maculabatis*	*Maculabatis macrura/gerrardi*	NRZ-Fisch-2023-007
	81	Carcharhinidae	*Carcharhinus*	*Carcharhinus sealei*	NRZ-Fisch-2023-008
	82	Carcharhinidae	*Carcharhinus*	*Carcharhinus sealei*	NRZ-Fisch-2023-009
	83	Hemiscylliidae	*Chiloscyllium*	*Chiloscyllium punctatum*	NRZ-Fisch-2023-010
	84	Hemiscylliidae	*Chiloscyllium*	*Chiloscyllium punctatum*	NRZ-Fisch-2023-011
Market samples	A	Gadidae	*Gadus*	*Gadus morhua*	NRZ-Fisch-2023-002a
	B	Salmonidae	*Salmo*	*Salmo salar*	NRZ-Fisch-2023-003a

### 2.5 DNA extraction

DNA was isolated with either a CTAB method (Rehbein, 2005) or with commercial DNA extraction kits (DNeasy Blood & Tissue Kit (Qiagen, Hilden, Germany); E.Z.N.A. Tissue DNA Kit (Omega Bio-tek, Norcross, GA, USA); NucleoSpin Food Mini Kit (Macherey-Nagel, Düren, Germany)) following the manufacturers' instructions. DNA concentrations and purities were measured photometrically or fluorometrically using a Qubit dsDNA Broadrange Kit (ThermoFisher) with a microvolume spectrophotometer (DS-11 FX, deNovix, Wilmington, DE, USA). DNA concentrations were adjusted to 10 ng/μl.

### 2.6 PCR amplification

The gene markers *cytb* (approx. 464 bp) and 16S rDNA (approx. 600 bp) and an additional pUC57 vector DNA region (542 bp) were amplified in triplex PCRs with the following forward and biotin-labelled reverse primers L14735 (5′-AAAAACCACCGTTGTTATTCAACTA-3′) and H1514AD-Biotin (5′-biotin-GCICCTCARAATGAYATTTGTCCTCA-3′), 16S-h (5′-CGCCTGTTTATCAAAAACAT-3′) and 16S-r-Biotin (5′- biotin-CCGGTCTGAACTCAGATCACGT-3′) as well as pUC57forw (5′-GATACGGGAGGGCTTACCA-3′) and pUC57rev-Biotin (5′-biotin-GCGCGGTATTATCCCGTATT-3′) as described before (Kappel et al., 2020). PCR products with biotin labels at the 5′-end of the forward DNA strands were amplified as well for both gene markers *cytb* and 16S rDNA. Primers were manufactured by Metabion, Planegg, Germany.

### 2.7 Microarray analysis

Oligonucleotide probes (Table 1 and Table B.1) with 3‘-C7 amino modification (manufactured by Metabion, Planegg, Germany) were spotted in triplicates with a concentration of 15 μM (150 pl per spot) onto the bottom of 8-well stripes with aldehyde modification (one microarray per well) by INTER-ARRAY by fzmb GmbH (Bad Langensalza, Germany). The DNA probes were spotted contact-less using piezoelectric dispensing technology as 19 × 19 arrays with 200 μm space between the spots. Correct spotting was checked by in-process controls. Additional antibody-spots for the enzyme horseradish peroxidase serve as staining controls and hereby define the array layout of the spotted DNA probes. For hybridization of the generated PCR amplicons on the prepared microarrays the INTER-ARRAY Hybridization Kit (INTER-ARRAY by fzmb GmbH) was used according to manufacturer's specifications. Briefly, PCR products were denatured for 5 min at 95 °C in a heat block and then immediately placed on ice. After washing and prehybridization of the arrays, the denatured PCR products were hybridized in a final dilution of 1:500 in hybridization buffer (10 μl diluted PCR product in a final volume of 100 μl) on the microarrays for one hour at 45 °C, except in the pre-tests where different temperatures and dilutions were tested. Afterwards, the arrays were washed, incubated with streptavidin-horseradish peroxidase conjugate and were finally stained as recommended by the manufacturer. The arrays were measured directly after staining in the INTER-VISION Reader. Arrays were processed using the INTER-VISION GENOTYPING 1.2.0 software (INTER-ARRAY by fzmb GmbH). Normalized signal intensities in arbitrary units were calculated automatically by dividing the average intensity of the automatically detected spot by the intensity of the automatically detected local background and ranged from 0 (no signal) to 1 (maximum signal). Hybridization data was analyzed using R version 4.2.3 in R studios (R Core Team, 2023; RStudio Team, 2023). Mean values of signal intensities were calculated for probe triplicates (Schauberger & Walker, 2021). If individual spots displayed poor qualities (*e.g.* irregular spot shapes indicating staining artefacts), values were excluded from calculation. Signal pattern specificity for particular fish species was assessed by comparing the signal intensities of all probes from all tested fish specimens with a Ward hierarchical clustering analysis (Murtagh & Legendre, 2014) using Euclidean or supremum distances and pattern similarity was displayed as a dendrogram (de Vries & Ripley, 2022).

## 3 Results and discussion

In this study a new approach of using universal DNA probes instead of species-specific probes was conducted to establish a DNA microarray for fish species identification (Huan et al., 2021; Jin et al., 2019; Kappel et al., 2020; Kappel et al., 2021; Xiang et al., 2022). The overall idea to design and select these universal DNA probes is depicted in Fig. 1 (compare with 2.2 and 2.3): A large fish set of 28,000 DNA sequences was processed on the one hand to design thousands of potential probe candidates and on the other hand to perform an *in silico* test for final probe selection. The final 96 probes plus the four control probes are shown in Table 1 (and Table B.1). Of these probes, 85 DNA probes targeted four more variable subregions of the *cytb* segment used for PCR amplification and 11 DNA probes targeted three more conserved 16S rDNA subregions (see Fig. A.1 and A.2 for probe binding positions).

### 3.1 Optimization of the DNA microarray assay conditions

To assess whether species-specific DNA probe signals can be obtained using this method and differentiate between fish species, assay conditions were first optimized. Four fish species with two individuals each were analyzed: *Gadus macrocephalus* (Pacific cod), *Gadus morhua* (Atlantic cod), *Pleuronectes platessa* (European plaice) and *Platichthys flesus* (European flounder) (Table 2). These were selected because (1) they represented two closely-related species each of two fish families and (2) the predicted number of DNA probe hybridizations were relatively high for *G. macrocephalus* and *G. morhua*, but relatively low for *P. platessa* and *P. flesus*. DNA of these individuals were amplified twice with either a biotin-labelled reverse primer (resulting in a biotin-labelled strand with orientation identical to the DNA probes) or a biotin-labelled forward primer (resulting in a biotin-labelled strand with orientation complementary to the DNA probes) and were tested on the DNA microarrays. Both biotin-labelled PCR product strands of a fish specimen showed no significant difference in DNA probe signal intensities (data not shown). It is assumed that in the case of using the biotin-labelled reverse strand, the forward strand of the denatured PCR product hybridizes to the DNA probes and the biotin-labelled reverse strand of the PCR products hybridizes to the forward strand resulting in triplex binding which may introduce an increased specificity to the assay (Frank-Kamenetskii & Mirkin, 1995; Giovannangeli et al., 1991; Jain, Wang, & Vasquez, 2008). Therefore, the biotin-labelled reverse primer was chosen to amplify all further PCR products in order to achieve a higher DNA probe signal pattern specificity.

**Fig. 2:**
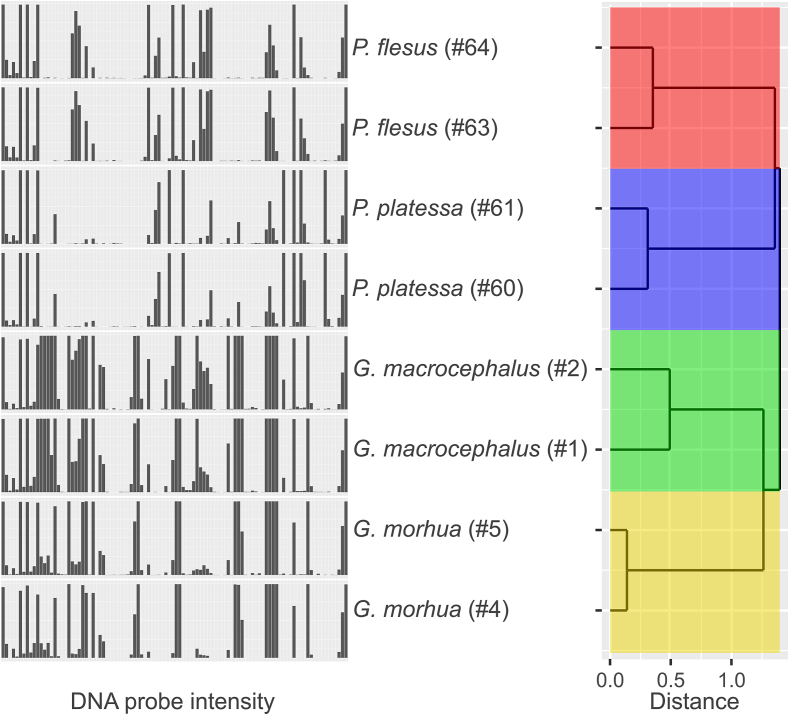
DNA probe signal patterns and supremum distances for the tested samples for assay optimization. Bar plots on the left show DNA probe intensities (order from left to right according to the DNA probe table from top to bottom from Table 1 and Table B.1) for *P. flesus* (#64, #63), *P. platessa* (#61, #60), *G. macrocephalus* (#2, #1) and *G. morhua* (#5, #4). Hierarchical clustering analysis using supremum distances of the signal patterns of the tested fish samples is displayed on the right as dendrogram. Fish species are indicated by colors. 45 °C for hybridization and a PCR product dilution of 1:500 were chosen as standard conditions.

DNA probe signal intensities depend among other factors on hybridization temperature and DNA concentration or PCR product dilution (Letowski, Brousseau, & Masson, 2004; Wang et al., 2005). Therefore, three different hybridization temperatures (40 °C, 45 °C and 50 °C) and three PCR product dilutions (1:125, 1:250 and 1:500) were examined. For each assay condition tested, the optimal assay conditions were chosen by comparing the signal intensities of all probes from all tested fish specimens with a hierarchical clustering analysis using supremum distances (Fig. 2 and Table B.2, B.4). The greatest intraspecific distance and the smallest interspecific distance were determined for each assay condition tested and the absolute value of the overall difference between these distances was calculated. In the same manner, the sum of intraspecific distances and the sum of interspecific distances were calculated and the absolute values of the overall differences between these distances were calculated. The greatest distance between intraspecific und interspecific distances (22.17 and 0.39, respectively) was observed with a hybridization temperature of 45 °C and a PCR product dilution of 1:500 (see also Table 3). In other words, using this hybridization condition, the difference between DNA probe signal patterns of individuals of the same species is minimized and the difference between DNA probe signal patterns of individuals of closely-related species is maximized at the same time (Fig. 2 and Table B.2, B.4).

**Table 3:** Supremum distances between the probe signal patterns of the tested fish samples for assay optimization. The greatest intraspecific distance and smallest interspecific distance were determined for each assay condition and the absolute values of the overall differences between these distances were calculated. In the same manner, the sum of intraspecific distances, the sum of interspecific distances and the absolute value of the overall difference between these distances were calculated.

Temperature	40 °C			45 °C			50 °C		
Dilution	1:125	1:250	1:500	1:125	1:250	1:500	1:125	1:250	1:500
Sum of intraspecific distance	1.96	2.14	1.34	1.64	1.54	1.29	1.54	1.45	1.86
Sum of interspecific distance	22.57	22.63	23.20	22.70	23.06	23.46	22.00	22.68	23.19
Absolute value of difference	20.61	20.50	21.86	21.06	21.52	**22.17**	20.47	21.24	21.33
Greatest intraspecific distance	0.77	0.82	0.48	0.48	0.56	0.49	0.61	0.74	0.81
Smallest interspecific distance	0.55	0.62	0.83	0.67	0.75	0.88	0.35	0.54	0.71
Absolute value of difference	0.22	0.21	0.35	0.20	0.19	**0.39**	0.26	0.20	0.10

### 3.2 Performance of the DNA probe set

**Fig. 3:**
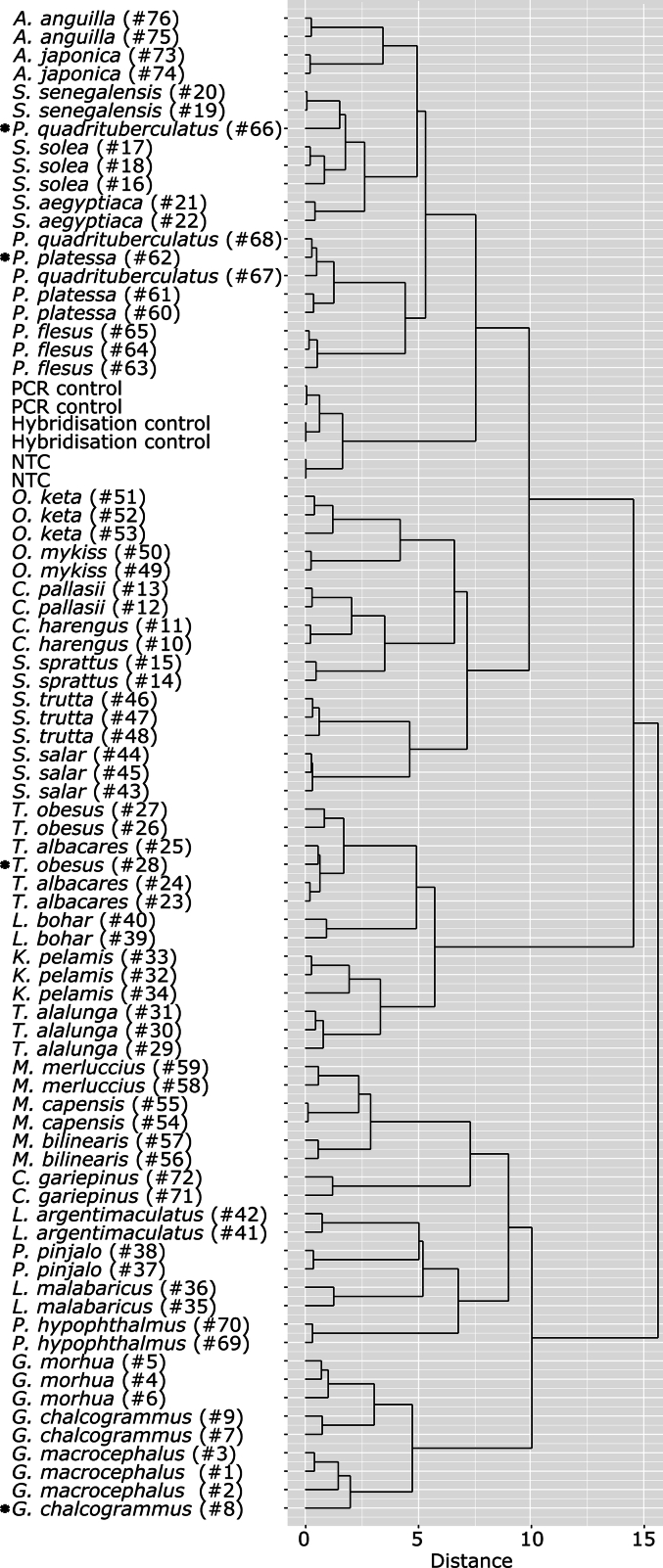
Distances of DNA probe signal patterns of Osteichthyes fish samples displayed as a dendrogram. Hierarchical clustering analysis using Euclidean distances of corresponding fish samples and assay controls is displayed. Sample IDs are written in brackets. Inconsistently clustered samples are indicated with asterisks.

To investigate the ability of the designed DNA probe set to generate species-specific signal patterns for various fish species, a total of 84 specimens belonging to bony fish species (Osteichthyes) and cartilaginous fish species (Chondrichthyes) were used as test samples. For Osteichthyes, 76 samples belonging to 31 fish species of the following 11 relevant fish families were analyzed with the universal DNA microarray: Anguillidae, Claridae, Clupeidae, Gadidae, Lutjanidae, Merlucciidae, Pangasiidae, Pleuronectidae, Salmonidae, Scombridae and Soleidae. PCR products from 2 to 3 individuals of each species were hybridized using the assay conditions considered as best (45 °C for hybridization and a PCR product dilution of 1:500). Species discrimination was assessed by comparing the signal intensities of all probes from all tested Osteichthyes fish specimens with a hierarchical clustering analysis using Euclidean distances (Fig. 3 and Table B.3, B.5). All specimens were additionally assessed with a hierarchical clustering analysis using supremum distances (Fig. A.3 and Table B.3, B.6), showing similar results. Individuals of the same species are clustered together in the dendrograms (Fig. 3 and Fig. A.3, S6). Only four samples out of 76 samples (5.3 %) are clustered inconsistently with other fish species: *Pleuronectes quadrituberculatus* (Alaska plaice) (#66) is not clustered with fish samples of the same species, but with two individuals of *Solea senegalensis* (Senegalese sole) (#19, #20), and *P. platessa* (#62) is clustered with two individuals of *P. quadrituberculatus* (#67, #68) (Fig. 3). *Thunnus obesus* (Atlantic Bigeye Tuna) (#28) is found in the cluster of *Thunnus albacares* (Pacific Yellowfin Tuna) (#23–25), and *Gadus chalcogrammus* (Alaska pollock or walleye pollock) (#8) is found in the cluster of *G. macrocephalus* (#1–3). DNA sequence analysis of *P. quadrituberculatus* (#66), *P. platessa* (#62) and *G. chalcogrammus* (#8) showed a few sequence differences between individuals of the same species at positions targeted by the DNA probes, presumably resulting in different DNA signal patterns and Euclidean and supremum distances (data not shown). In the case of *T. obesus* (#28) weak PCR product signals in contrast to all other samples were observed, indicating potential degradation of the extracted DNA. Less PCR product amount could have had an effect on the hybridization event, resulting in different DNA probe signals (Wang et al., 2005). Overall, species-specific DNA signal patterns were obtained for all analyzed fish species. Still, more replicates of samples and many more fish species, families and individuals need to be tested to validate the method further.

**Fig. 4:**
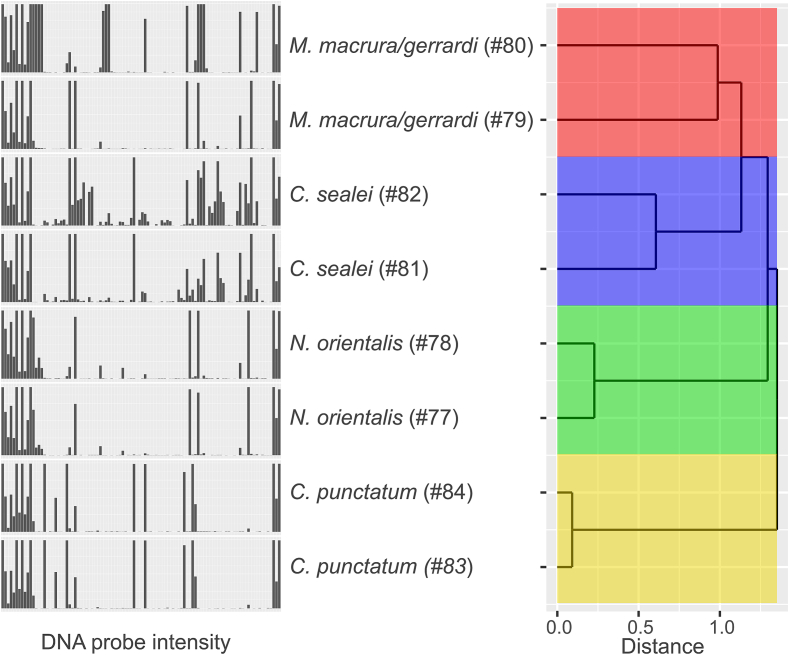
DNA probe signal patterns and dendrogram for Chondrichthyes. Bar plots on the left show DNA probe intensities (order from left to right according to the DNA probe table from top to bottom from Table 1 and Table B.1) for *M. macrura*/gerrardi (#80, #79), *C. sealei* (#82, #81), *N. orientalis* (#78, #77) and *C. punctatum* (#84, #83). Hierarchical clustering analysis using supremum distances of corresponding fish species is displayed on the right as dendrogram. Fish species are indicated by same colors.

To demonstrate the broad applicability of the DNA microarray with more exotic fish species, four fish species of sharks and rays (Chondrichthyes) with two individuals each were analyzed: *Maculabatis macrura/gerrardi* (long-tailed or whitespotted whipray, exact species identification neither morphologically nor genetically possible (Clark-Shen et al., 2021)), *Neotrygon orientalis* (oriental bluespotted maskray), *Carcharhinus sealei* (blackspot shark) and *Chiloscyllium punctatum* (demersal brown banded bamboo shark). Species discrimination was assessed again by comparing the signal intensities of all probes from all tested Chondrichthyes fish specimens with a hierarchical clustering analysis using supremum distances (Fig. 4, Table B.3, B.7). Even though both individuals of the analyzed species are clustered together (Fig. 4 on the right), the DNA probe signal patterns for individuals of the same species sometimes differed (Fig. 4 on the left). Both specimens of *N. orientalis* and *C. punctatum* each, are clustered together and showed a very similar species-specific signal pattern. However, the signal patterns of both samples of *C. sealei* differed slightly more, and specimens of *M. macrura/gerrardi* displayed even greater differences in signal patterns. As also the DNA sequences of the analyzed *cytb* segment varied by 33 nucleotides and the COI segment varied by 32 nucleotides (data not shown), it is very likely, that one sample (#80) is *M. gerrardi* and the other sample (#79) is *M. macrura*.

The universal DNA microarray featured three assay controls that allow an automatic troubleshooting procedure (Huan et al., 2021; Kappel et al., 2020; Kappel et al., 2021; Tortajada-Genaro et al., 2011). The DNA microarray was checked with pure water (no-template control, NTC), with the synthetic oligonucleotide target (hybridization control) and with the oligonucleotide target combined with amplified pUC57 vector (DNA PCR control). All three controls were tested twice and DNA signal intensities were analyzed together with all previously tested Osteichthyes fish specimens with a hierarchical clustering analysis using supremum distances (Fig. 3 and Table B.3, B.7). All control microarrays exhibited the expected DNA probe signals and were clustered respectively (hybridization control, PCR control and NTC in Fig. 3).

### 3.3 Proof of concept with market samples

**Fig. 5:**
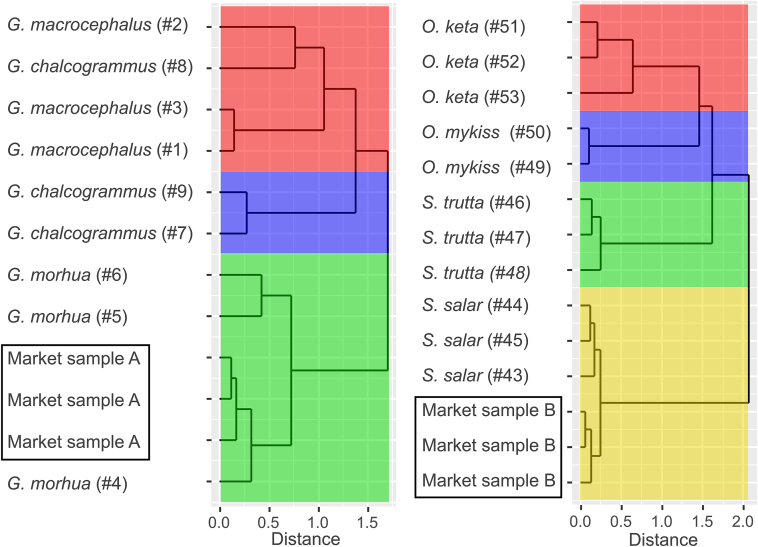
Analysis of two market samples. Both market samples were measured in triplicate and analyzed with all previous fish samples in a hierarchical clustering analysis. Supremum distances are only shown for market samples and species-related corresponding fish species as dendrograms and particular fish species are indicated by colors (except for #8). Market sample A labelled as “Atlantic cod, *Gadus morhua*” is shown on the left and market sample B labelled as “Atlantic salmon, *Salmo salar*” is analyzed on the right.

To illustrate the applicability of the universal DNA microarray, two samples from market-relevant species were analyzed with the universal DNA microarray: Market sample A was labelled as “Atlantic cod, *Gadus morhua*” and market sample B was labelled as “Atlantic salmon, *Salmo salar*”. Both species were analyzed in triplicate and species identification was performed by comparing the signal intensities of each market sample with the signal patterns of all previously analyzed authentic (reference) fish specimens with a hierarchical clustering analysis using supremum distances. Sections of the dendrograms displaying the market samples together with the reference specimens with the most similar signal patterns are shown in Fig. 5 (see also Table B.3, B.8A, B.8B). Market sample A (“Atlantic cod, *Gadus morhua*”) was clustered with specimens of *G. morhua* (#4–6) (Fig. 5 on the left) and market sample B (“Atlantic salmon, *Salmo salar*”) was clustered with specimens of *S. salar* (#43–45) (Fig. 5 on the right). DNA barcoding of both market samples verified the results of the universal DNA microarray.

## 4 Conclusion

As a proof-of-principle a universal DNA microarray was developed with 100 DNA probes (96 newly *in silico* designed probes and four control probes) that reliably generated species-specific DNA probe signals for all 86 fish samples and eight control assays. The microarray differs from existing methods in that it does not require perfect matches in any chemical sense. Instead, the use of hybridization patterns implements a best-match philosophy. Nearly every sample tested was correctly clustered with the other samples of the same species. No doubt, exceptions and weaknesses will be found with the current probe set. There is also room for improvement in both computational and chemical aspects. The computational steps might be seen as rather *ad hoc* and there is room for automation. Probe sequences were chosen with simple matching criteria. This should be formalized and complemented by criteria to reject unwanted matches.

This study should serve as a proof-of-principle and further validation should be carried out. More replicates and species should be analyzed to confirm current results. Likewise, the robustness of the DNA array should be determined, e. g. by using different thermocycler or users and laboratories. The set of 100 DNA probes could even be applied to other types of DNA chips. However, the analysis of two market samples that are highly relevant to European consumers illustrates how well the universal DNA microarray already works. This method can easily be applied in molecular laboratories and will be a useful tool for species identification of fishery products due to its speed, versatility and automatic data analysis. Such a universal DNA microarray for fish species authentication has not previously been demonstrated and could play an important role in fighting food fraud and adulteration.

## Funding

This IGF Project of the FEI is supported *via* AiF within the programme for promoting the Industrial Collective Research (IGF) of the Federal Ministry of Economic Affairs and Climate Action (BMWK), based on a resolution of the German Parliament (project number 01IF21952N).

## Ethical approval

This study does not contain experiments that involve human or living animals. The authors used material from deceased fish from the commercial fishing industry that had been killed by the commercial fishing industry for the food supply chain.

## CRediT authorship contribution statement

**Patrizia Bade:** Writing – original draft, Visualization, Validation, Investigation, Formal analysis, Data curation. **Sebastian Stix:** Writing – review & editing, Software, Investigation, Formal analysis, Data curation. **Kristina Kappel:** Writing – review & editing, Supervision, Project administration, Funding acquisition, Conceptualization. **Jan Fritsche:** Writing – review & editing, Funding acquisition, Conceptualization. **Ilka Haase:** Writing – review & editing, Funding acquisition, Conceptualization. **Andrew Torda:** Writing – review & editing, Supervision. **Nils Wax:** Writing – review & editing. **Markus Fischer:** Writing – review & editing, Conceptualization. **Dirk Brandis:** Writing – review & editing, Supervision. **Ute Schröder:** Writing – review & editing, Supervision, Project administration, Funding acquisition, Conceptualization.

## Declaration of competing interest

The authors declare that they have no known competing financial interests or personal relationships that could have appeared to influence the work reported in this paper.

## Data Availability

Data will be made available on request.

## Appendix A Supplementary data

Supplementary material 1 **Appendix A:** **Fig. A.1**: DNA sequence alignment with representative fish DNA sequences and 85 designed DNA probes targeting four more variable subregions of the *cytb* fragment (visualized in Jalview). **Fig. A.2**: DNA sequence alignment with representative fish DNA sequences and 11 designed DNA probes targeting three more conserved subregions of the 16S rDNA fragment (visualized in Jalview). **Fig. A.3**: Distances of DNA probe signal patterns of Osteichthyes fish samples displayed as a dendrogram. Hierarchical clustering analysis using supremum distances of corresponding fish samples and assay controls is displayed. Sample IDs are written in brackets. Inconsistently clustered samples are indicated with asterisks. Supplementary material 1

Supplementary material 2 **Appendix B:** **Table B.1**: Sequences and characteristics of all 100 universal DNA probes (96 newly designed DNA probes and four control probes (Kappel, 2009)) **Table B.2**: DNA microarray measurement data for assay optimization **Table B.3**: Data of DNA microarray measurements of all tested samples **Table B.4**: Matrix of supremum distances between DNA probe signal patterns of all tested samples for assay optimization **Table B.5**: Matrix of Euclidean distances between DNA probe signal patterns of all tested Osteichthyes fish samples **Table B.6**: Matrix of supremum distances between DNA probe signal patterns of all tested Osteichthyes fish samples **Table B.7:** Matrix of supremum distances between DNA probe signal patterns of all tested Chondrichthyes fish samples **Table B.8 A:** Matrix of supremum distances between DNA probe signal patterns of the market sample A labelled as “Atlantic cod, *Gadus morhua*” and closely-related species **Table B·8B:** Matrix of supremum distances between DNA probe signal patterns of the market sample B labelled as “Atlantic salmon, *Salmo salar*” and closely-related species Supplementary material 2
