# Supplementary material for: A universal DNA microarray for rapid fish species authentication
Source: Food Chem (Oxf). 2025 Jan 19;10:100241. doi: 10.1016/j.fochms.2025.100241 (PMC11795077; doi:10.1016/j.fochms.2025.100241)
Supplement: Supplementary material 1 — Appendix A: Fig. A.1: DNA sequence alignment with representative fish DNA sequences and 85 designed DNA probes targeting four more variable subregions of the cytb fragment (visualized in Jalview). Fig. A.2: DNA sequence alignment with representative fish DNA sequences and 11 designed DNA probes targeting three more conserved subregions of the 16S rDNA fragment (visualized in Jalview). Fig. A.3: Distances of DNA probe signal patterns of Osteichthyes fish samples displayed as a dendrogram. Hierarchical clustering analysis using supremum distances of corresponding fish samples and assay controls is displayed. Sample IDs are written in brackets. Inconsistently clustered samples are indicated with asterisks. [file mmc1.docx]

Supporting Information

**All-fish chip: A universal DNA microarray for rapid fish species authentication**

Patrizia Bade^1^, Sebastian Stix^2^, Kristina Kappel^1*^, Jan Fritsche^2^ , Ilka Haase^3^, Andrew Torda^4^, Nils Wax^5^, Markus Fischer^5^, Dirk Brandis^6^, Ute Schröder ^2^

^1^ National Reference Centre for Authentic Food, Max Rubner-Institut (MRI), Hermann-Weigmann-Str. 1, 24103, Kiel, Germany

^2^ Department of Safety and Quality of Milk and Fish Products, Max Rubner-Institut (MRI), Hermann-Weigmann-Str. 1, 24103, Kiel, Germany

^3^ National Reference Centre for Authentic Food, Max Rubner-Institut (MRI), E.-C.-Baumann-Str. 20, 95326, Kulmbach, Germany

^4^ Centre for Bioinformatics, University of Hamburg, Albert-Einstein-Ring 8, 22761 Hamburg, Germany

^5^ Hamburg School of Food Science, Institute of Food Chemistry, University of Hamburg, Grindelallee 117, 20146, Hamburg, Germany

^6^ Zoological Museum of the University of Kiel, Hegewischstraße 3, 24105 Kiel, Germany

^*^Correspondence: Kristina Kappel


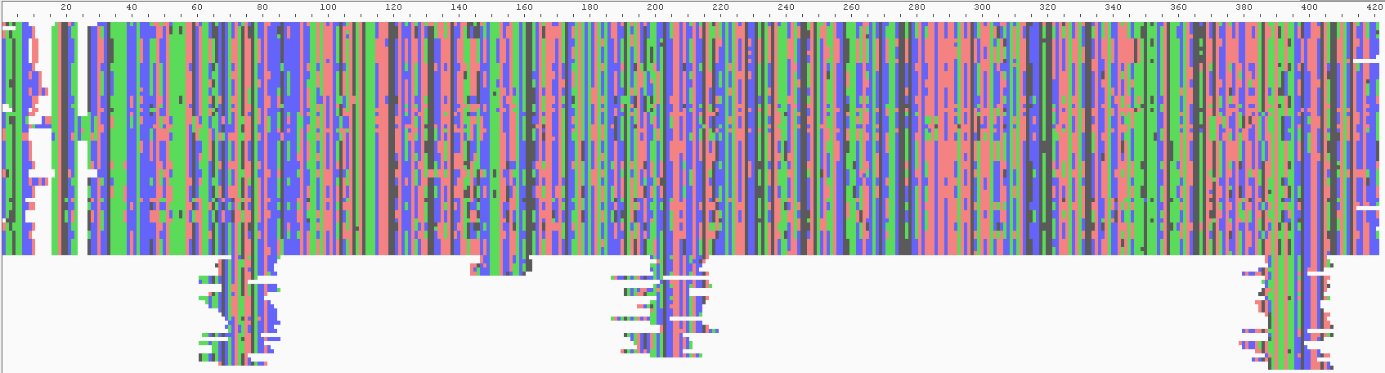


Figure A.1: DNA sequence alignment with representative fish DNA sequences and 85 designed DNA probes targeting four more variable subregions of the *cytb* fragment (visualized in Jalview).


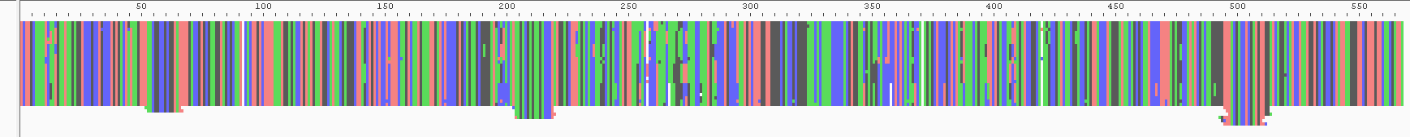


Figure A.2: DNA sequence alignment with representative fish DNA sequences and 11 designed DNA probes targeting three more conserved subregions of the 16S rDNA fragment (visualized in Jalview).


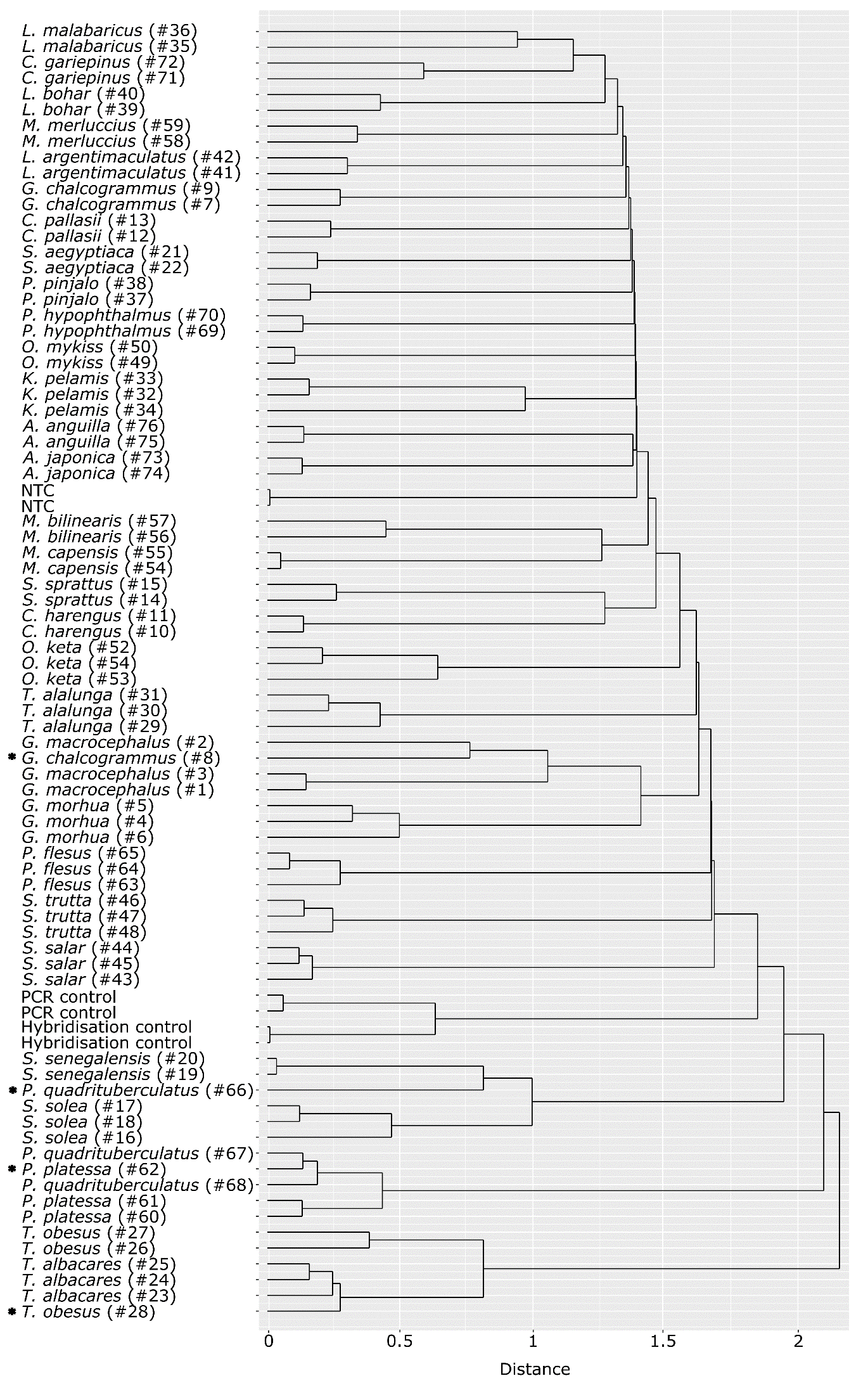


Figure A.3: Distances of DNA probe signal patterns of Osteichthyes fish samples displayed as a dendrogram. Hierarchical clustering analysis using supremum distances of corresponding fish samples and assay controls is displayed. Sample IDs are written in brackets. Inconsistently clustered samples are indicated with asterisks.
